# The prominent role of a CDR1 somatic hypermutation for convergent IGHV3-53/3-66 antibodies in binding to SARS-CoV-2

**DOI:** 10.1080/22221751.2022.2063074

**Published:** 2022-04-25

**Authors:** Xiaolong Tian, Xiaoyi Zhu, Wenping Song, Zhenlin Yang, Yanling Wu, Tianlei Ying

**Affiliations:** a MOE/NHC Key Laboratory of Medical Molecular Virology, School of Basic Medical Sciences, Shanghai Medical College, Fudan University, Shanghai, People’s Republic of China; b Shanghai Engineering Research Center for Synthetic Immunology, Shanghai, People’s Republic of China; c Department of Pulmonary Medicine, Zhongshan Hospital, Fudan University, Shanghai, People’s Republic of China; d Shanghai Key Laboratory of Lung Inflammation and Injury, Shanghai, People’s Republic of China

**Keywords:** SARS-CoV-2, monoclonal antibody, somatic hypermutation, IGHV3-53/3-66, antibody affinity

## Abstract

In the fight against severe acute respiratory syndrome coronavirus-2 (SARS-CoV-2), monoclonal antibodies (mAbs) serve as key strategies for the rapid prevention and treatment of COVID-19. However, analysis to fully characterize functional SARS-CoV-2 mAbs is still needed. In this study, by interrogating 1,695 published or patented mAbs of human origin and validated SARS-CoV-2-binding potency, we found a highly preferential usage of IGHV3-53/3-66 germline genes that was then revealed as a distinct selectivity of SARS-CoV-2-induced humoral immunity across other coronaviruses. Moreover, among the rare somatic hypermutations, we identified a novel mutation signature of F27 to I, L, or V with high frequency, which was located in the CDR1 region of the heavy chain among IGHV3-53/3-66-encoded antibodies. This convergent mutation contributed to improving SARS-CoV-2 binding affinity and may advance our knowledge of the humoral immunity to SARS-CoV-2.

Since the outbreak of coronavirus disease 2019 (COVID-19) in late 2019, unprecedented efforts have been devoted to developing therapeutic interventions to mitigate the pandemic, among which monoclonal antibodies (mAbs) that potently bind to and neutralize severe acute respiratory syndrome-coronavirus 2 (SARS-CoV-2), the causative agent of COVID-19, provide an attractive treatment strategy [1]. A total of 10 vaccines have been approved for use by WHO to date (https://covid19.trackvaccines.org/agency/who/), yet the molecular features that contribute to an effective antibody response are not entirely clear. For this purpose, based on the antibody collections screened by each lab, researchers have described features shared by SARS-CoV-2 functional mAbs [2–5], providing key insights into the molecular basis of B cell response to SARS-CoV-2 infection and facilitating rational vaccine design. However, due to the limited quantity and source of the acquired mAbs, larger-scale sequence analysis of functional SARS-CoV-2 mAbs with various origins needs to be carried out to find more convincing and novel signatures among all the published SARS-CoV-2 mAbs.

To comprehensively analyse the characteristics of SARS-CoV-2 functional antibodies and better understand the humoral immunity to SARS-CoV-2, we retrieved all entries collected before 6 June 2021, from CoV-AbDab [6], a database that documents all published or patented antibodies binding to coronaviruses (http://opig.stats.ox.ac.uk/webapps/covabdab/). After filtering for human-origin SARS-CoV-2-binding mAbs with the information of immunoglobulin heavy (IGHV) and light (IGLV or IGKV) variable germline gene usage being provided, 1,695 mAbs with validated binding potency were identified and included for further analysis. We then quantified the frequency of germline gene usages and found that the top 6 most used heavy chain germlines were IGHV3-53 (8.83%), IGHV3-30 (8.83%), IGHV1-69 (7.83%), IGHV3-66 (6.12%), IGHV3-30-3 (5.24%) and IGHV3-23 (4.53%) (supplementary Fig. S1); Among them, IGHV3-53 and the closely related IGHV3-66 (only a substitution of Ile13Val occurred within framework region 1) together constitute 14.95% of SARS-CoV-2-binding antibodies. Further, the paired light chains of these IGHV genes were scattered (Figure 1a), suggesting that the identity of the heavy chain, rather than the light chain, was critical for the SARS-CoV-2 interaction. Interestingly, it was observed that phylogenetically close heavy chains tend to possess similar light chain pairing modes (Figure 1b). By interrogating a total of 500 cross-reactive antibodies (namely antibodies that can bind to SARS-CoV-2 and at least one more coronavirus) gathered in filtered mAbs collection, a remarkable shrink was found in the germline gene usages of IGHV3-53 (1.4%) and IGHV3-66 (0.06%), while IGHV1-69, IGHV3-30, IGHV3-30-3, and IGHV3-23 were still used dominantly, taking up 17.2%, 14.4%, 5.6% and 3.8% of total cross-reactive antibodies, respectively (supplementary Fig. S1). Taken together, these results indicate that IGHV genes and the overall usage patterns of the paired light chain have been preferentially selected by SARS-CoV-2 infection. Notably, IGHV3-53/3-66-mediated antibody response is largely confined by the specific immunogenicity of SARS-CoV-2 among all of the coronaviruses.

**Figure 1.**
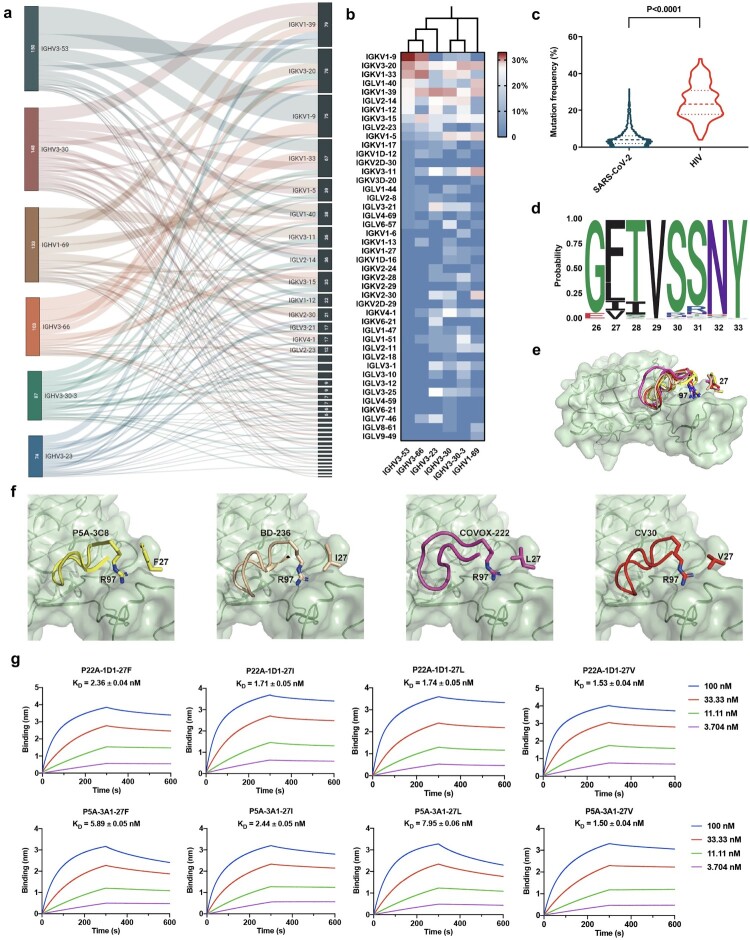
Characteristic analysis of SARS-CoV-2-binding human monoclonal antibodies. (a) Sankey diagram showing the pairing information between the top 6 most used heavy chain genes and the corresponding light chain genes in SARS-CoV-2-binding antibodies. The number of antibodies originated from each gene is indicated in the left column. (b) Light chain pairing modes of the top 6 most used IGHV genes in SARS-CoV-2-binding antibodies as shown in the heatmap. The phylogenetic neighbor-joining tree among IGHV germline genes is displayed on the top of the heatmap. (c) Violin plot comparing somatic hypermutation rates of SARS-CoV-2- and HIV-binding antibodies. The numbers of antibodies included for the analysis were 1447 for SARS-CoV-2 and 237 for HIV. The quartiles are indicated by dashed lines. The *p* value was calculated by two-tailed Student’s t test. (d) Sequence logos for the CDR1 regions of 217 IGHV3-53/3-66-binding antibodies. The position of each residue is labelled on the *x*-axis based on IMGT numbering. The occurrence probability of each amino acid is labelled on the *y*-axis. (e-f) Interactions between CDR3 of four representative IGHV3-53/3-66-binding antibodies and SARS-CoV-2 RBD and zoom-in of P22A-1D1 (PDB entry 7CHS), BD-236 (PDB entry 7CHB), COVOX-222 (PDB entry 7NX6) and CV30 (PDB entry 6XE1). The RBD is deciphered as green ribbon and surface. Elements from P22A-1D1, BD-236, COVOX-222, and CV30 are coloured in yellow, brown, magenta, and red, respectively. CDR3 is presented as cartoon. R97 and residues on position 27 are presented as sticks. (g) Binding kinetics of mAb P22A-1D1, P5A-3A1 and their 27I, 27L and 27 V variants to SARS-CoV-2 RBD measured by BLI. The mean ± SD from three independent experiments are shown.

Germline mAbs, denoted here as antibodies without any somatic hypermutation (SHM) occurred in the IGHV, were frequently detected in the SARS-CoV-2 functional antibody collection (supplementary Table S1). Their SHM frequencies were thus calculated for dissecting features of the acute infectious pathogen. To this end, all the 1,447 mAbs, acquired with full-length heavy chain sequences, were assigned to the inferred germline IGHV protein sequences by IgBlast [7]. The mutation frequencies that appeared in the whole variable region were computed by SHazaM [8]. As a result, the collection of SARS-CoV-2-binding mAbs exhibited an average of 4.93% amino acid mutation rate (Figure 1c), which was comparable to the SHM frequencies in healthy individuals’ IgG repertoires [9, 10]. As a contrast, sequences of 237 antibodies against the chronic infectious pathogen human immunodeficiency virus (HIV) from bNAber [11], a database of broadly neutralizing HIV antibodies, were calculated for their SHM frequencies. These chronic infection-specific mAbs displayed a much higher amino acid mutation frequency of 24.82% (Figure 1c). Therefore, it seems that mAbs without sufficient SHM were elicited to urgently and effectively confront the acute SARS-CoV-2 infection.

To further explore the signatures of the exiguous SHM within the functional SARS-CoV-2 mAb collection, we aligned the heavy chain sequences to the corresponding germline V and J genes and found two concentrated mutations among IGHV3-53/3-66 antibodies. One of them was Y58F, which has been recently identified by Tan *et al* [2] from 214 published IGHV3-53/3-66 receptor-binding domain (RBD) antibodies. It was reported as a common SHM that improves the affinity of IGHV3-53/3-66 antibodies with a short complementarity-determining region 3 of the heavy chain (HCDR3) to RBD. Despite the loss of a hydrogen bond with the RBD, Y58F can form strong T-shaped π-π stacking interactions with the amide backbone of RBD T415. The other mutation was a novel common SHM identified by us from 217 IGHV3-53/3-66 RBD antibodies. Whereas residue 27 in the IMGT numbering scheme is Phe (F) in the germline antibodies, three hydrophobic residues, Ile (I), Leu (L), and Val (V), occupy this position at a frequency of 37.33% in these antibodies (Figure 1d).

To understand whether this common substitution played a role in their RBD recognition, we analyzed all the IGHV3-53/3-66 mAbs of those the structures have been parsed. Notably, almost all IGHV3-53/3-66 mAbs shared the same binding mode with RBD (supplementary Fig. S2). Among them, four representative mAbs, i.e. P5A-3C8 (PDB: 7CHP), BD-236 (PDB: 7CHB), COVOX-222 (PDB: 7NX6), and CV30 (PDB: 6XE1), which possessed F, I, L, and V at residue 27 respectively, were dissected separately to focus on the effect of mutations at residue 27 on antibody function (Figure 1e). Residue 27 locates in CDR1 of the variable heavy chain and is barely involved in the direct interaction with RBD. However, F27 forms a strong π-cation interaction with R97 (Figure 1c), which locates at the beginning of HCDR3 and is highly conserved in the IGHV3-53 antibodies (Figure 1f). It is evident that HCDR3 is critical to the recognition of antigen and the binding process is dynamic. The π-cation interaction between F27 and R97 may restrict the dynamic movement of HCDR3 by forming a steric hindrance. The substitution of aromatic residue F to hydrophobic I, L or V, which lacks the phenyl group, weakened the restriction and would result in higher flexibility of CDR3, likely facilitating interaction between HCDR3 of antibodies and RBD and enhancing the affinity of antibodies. Considering that the amino acid mutations of SARS-CoV-2 functional mAbs are not more than 5% on average, our results suggest that the high incidences of mutations from F to I, L, and V at residue 27 within IGHV3-53/3-66 mAbs might be meaningful in generating antibodies of higher affinities during the emergent response to SARS-CoV-2.

To further validate the functional role of F27 mutation, P22A-1D1 and P5A-3A1 [12], the only two fully germline IGHV3-53/3-66 mAbs equipped with structure information were selected to exclude the interference of mutations on the other residues. Both antibodies and their I27, L27 and V27 variants were expressed and purified (supplementary Fig. S3). The binding affinities of these 8 antibodies to SARS-CoV-2 RBD were then measured by bio-layer interferometry (BLI). Despite the high binding affinity of the pristine P22A-1D1 (2.36 nM) and P5A-3A1 (5.89 nM), the affinity of 5 of the 6 antibody variants was further improved by 1.36–3.93 folds (Figure 1g), dominantly caused by the slower process of dissociation. Moreover, the mutations had the potential to enhance the SARS-CoV-2 pseudovirus-neutralizing capacities of P22A-1D1 by 2.94-3.86 fold (IC_50_ of 0.544 μg/mL for 27F, 0.185 μg/mL for 27I, 0.141 μg/mL for 27L and 0.146 μg/mL for 27 V) (supplementary Fig. S4), although no significant elevation of neutralizing capacities was detected for the P5A-3A1 variants (supplementary Fig. S4). Furthermore, we generated germline formats of BD-236, COVOX-222, and CV30 at residue 27 by reverse mutation. It was found that binding affinities were decreased by 1.37, 2.02, and 5.14 folds, respectively (supplementary Fig. S5). Collectively, these results preliminarily demonstrate a beneficial effect of F27 to I, L or V mutations for IGHV3-53/3-66 mAbs on combating SARS-CoV-2, although more SARS-CoV-2-binding mAbs should be examined to confirm this finding.

Furthermore, to investigate whether double mutations significantly increase antibody affinity, we generated a panel of variants with single mutations at Y58 and double mutations at F27 and Y58 to P5A-3A1 and P22A-1D1, respectively, and tested their binding affinity by BLI. The results showed that either the single mutation of Y58F or F27 in P5A-3A1 and P22A-1D1 can improve the antibody affinity (Figure 1g and supplementary Fig. S6). Compared to the single-mutation, specially F27 variants, the double mutations (including Y58F and F27I/L/V) had higher binding affinity, suggesting simultaneous mutations at positions 58 and 27 may have synergistic function in increasing affinity.

In summary, our results confirmed that SARS-CoV-2 bears distinct selectivity in the IGHV gene usages, although it exhibits acknowledged hallmarks of the acute infectious pathogens in eliciting antibodies with extensive utilization of the IGHV1-69 gene and the rare occurrence of SHM. Notably, for the SARS-CoV-2-corresponding IGHV3-53/3-66-encoded antibodies of all coronaviruses, firstly identified herein, a novel and common F27 to I, L, or V mutation comprised in the CDR1 was found to play a prominent role in the effective antibody function, and may have synergy effect with Y58F mutation to improve antibody affinity. Therefore, this finding provides an explanation for the phenomenon that functional mAbs continue to potently bind and neutralize SARS-CoV-2 even though lacking sufficient affinity maturity. Our finding benefits the genetic engineering of SARS-CoV-2 antibodies, and emphasizes the timeliness of antibody screening in the large naive antibody library of healthy donors that lack SHM for confronting emerging acute infectious diseases.

## Supplementary Material

Supplemental Material
