# Supplementary material for: The prominent role of a CDR1 somatic hypermutation for convergent IGHV3-53/3-66 antibodies in binding to SARS-CoV-2
Source: Emerg Microbes Infect. 2022 Apr 25;11(1):1186–90. doi: 10.1080/22221751.2022.2063074 (PMC9045774; doi:10.1080/22221751.2022.2063074)
Supplement: Supplemental Material [file TEMI_A_2063074_SM3569.docx]

**Supplementary materials**

**The prominent role of a CDR1 somatic hypermutation for convergent IGHV3-53/ IGHV3-66 antibodies responding to SARS-CoV-2**

Xiaolong Tian^1†^, Xiaoyi Zhu^1†^, Wenping Song^1^, Zhenlin Yang^2,3,4^, Yanling Wu^1,2^**^*^**, Tianlei Ying^1,2^**^*^**

*^1^MOE/NHC Key Laboratory of Medical Molecular Virology, School of Basic Medical Sciences, Shanghai Medical College, Fudan University, Shanghai 200032, China;* [*xltian18@fudan.edu.cn*](mailto:xltian18@fudan.edu.cn) *(X.T.);* [*20111010095@fudan.edu.cn*](mailto:20111010095@fudan.edu.cn) *(X.Z.);* [*21111010037@fudan.edu.cn*](mailto:21111010037@fudan.edu.cn) *(W.S.);*

*^2^Shanghai Engineering Research Center for Synthetic Immunology, Shanghai 200032, China;* [*yang_zhenlin@fudan.edu.cn*](mailto:yang_zhenlin@fudan.edu.cn) *(Z.Y.);*

*^3^Department of Pulmonary Medicine, Zhongshan Hospital, Fudan University, Shanghai 200032, China;*

*^4^Shanghai Key Laboratory of Lung Inflammation and Injury, Shanghai 200032, China.*

*****To whom correspondence should be addressed: [tlying@fudan.edu.cn](mailto:tlying@fudan.edu.cn) (T.Y.); [yanlingwu@fudan.edu.cn](mailto:yanlingwu@fudan.edu.cn) (Y.L.W.)

†These authors share co-first authorship.

**Supplementary information include:**

Materials and Methods

Fig. S1 to S6

Tab. S1 to S2

**Materials and Methods**

***Cell lines***

Huh-7 cells and 293F cells were cultured in Dulbecco’s modified Eagle’s medium (DMEM) supplemented with 10% fetal bovine serum (FBS) in 37°C, 5% CO_2_ atmosphere.

***Bioinformatics analysis of antibody mutation frequency***

The protein sequences of two antibody collections were downloaded from the CoV-AbDab database (<http://opig.stats.ox.ac.uk/webapps/covabdab/>) for coronaviruses and bNAber database (<http://bNAber.org>) for HIV respectively. Protein sequences of “F+ORF+in-frame P” alleles were obtained as references from the IMGT (<http://www.imgt.org/vquest/refseqh.html#VQUEST>), to conduct sequence alignments for these mAbs using the igblastp module of IgBLAST.^1^ Next, the whole IGHV segment mutation frequencies of the two antibody collections were analyzed by SHazaM package,^2^ using the command of “observedMutations”.

***Three-dimension structural analysis***

All the structural representations were drawn by PyMOL (DeLano Scientific, San Carlos, CA, USA).^3^ The structures of RBD in complex with P22A-1D1 (Phe27, PDB entry 7CHS), B38 (Phe27, PDB entry 7BZ5), BD-629 (Phe27, PDB entry 7CH5), C1A-B3 (Phe27, PDB entry 7KFW), C1A-B12 (Phe27, PDB entry 7KFV), C1A-C2 (Phe27, PDB entry 7KFX), C1A-F10 (Phe27, PDB entry 7KFY), CC12.1 (Phe27, PDB entry 6XC2), COVA2-04 (Phe27, PDB entry 7JMO), LY-CoV4812 (Phe27, PDB entry 7KMI), P2B-1A10 (Phe27, PDB entry 7CZQ), P4A1 (Phe27, PDB entry 7CJF), P5A-1D2 (Phe27, PDB entry 7CHO), P5A-3C8 (Phe27, PDB entry 7CHP), BD-236 (Ile27, PDB entry 7CHB), BG4-25 (Ile27, PDB entry 7M6D), COVOX-222 (Leu27, PDB entry 7OR9), COVOX-269 (Leu27, PDB entry 7NEH), LY-CoV488 (Leu27, PDB entry 7KMH), COVOX-158 (Val27, PDB entry 7BEJ) and CV30 (Val27, PDB entry 6xe1), respectively, were aligned and rendered using PyMOL.

***Antibody expression and purification***

The genes encoding for P22A-1D1, P5A-3A1, BD-236, COVOX-222, and CV30 were synthesized by Genewiz (Suzhou, China) and cloned into pTT expression vectors. The I27, L27, and V27 variants in heavy chains of both antibodies were constructed using overlapping PCR. Briefly, the circular pTT vector was digested using BsmBI and SfiI restriction endonucleases (NEB, USA). Two complementary primers containing the desired mutation of heavy chain 27I, 27L, 27V and two flanking primers including restriction sites were designed (Tab. S2). For the construction of each variant, in the primary polymerase chain reaction (PCR) step, two complementary primers were used for amplifying two contiguous segments with two flanking primers separately. The two PCR products were cloned into the nicked vector with homologous recombination methods by a one-step PCR cloning kit (Novoprotein, China). Similar methods were adopted to add Y58F mutation to P22A-1D1 and P5A-3A1 as well as their variants and to construct 27F reverse mutation on BD-236, CV30 and COVOX-222 (Tab. S2). Followed by a transformation into E. coli TOP10 competent cells, the recombinant mutant plasmids were verified by Sanger sequencing. Then, the heavy and light chain vectors were co-transfected into 293F cells using the Expi293 expression system (Thermo Fisher Scientific) according to the manufacturer’s instructions. After 96 hours of expression, the cells were centrifuged at 3900 rpm for 15 min at 4℃ and the supernatant was collected and filtered with a 0.22 µm vacuum filter. Then, the proteins were purified using Protein A affinity chromatography (GE Healthcare) and dialyzed against phosphate-buffered saline (PBS). The purity and homogeneity of proteins were verified by SDS-PAGE and the protein concentrations were measured by NanoDrop 2000 (Thermo).

***Bio-layer interferometry (BLI) binding assays***

The binding kinetics of all the antibodies and their variants to SARS-CoV-2 RBD with his-tag was measured by BLI on an Octet-RED96 system (ForteBio). Briefly, the recombinant RBD in 10 μg/ml in kinetic buffer (PBS containing 0.05% Tween 20) was immobilized onto Ni-NTA biosensors (ForteBio) until saturation. Loaded biosensors were dipped into wells containing threefold serial dilutions antibodies in the kinetic buffer. The baseline was established in kinetic buffer. After antibody association with RBD for 300 seconds, the RBD: antibody complexes were allowed to dissociate in kinetics buffer for another second. Mean *k*_on_, *k*_off_, and K_D_ values were determined by averaging binding curves within a dilution series having R^2^ values greater than 95% confidence level. Graph was made using Prism 8 (GraphPad).

***Neutralization assay***

Pseudotyped viruses incorporated with spike protein of SARS-CoV-2 were prepared. 293 T cells were co-transfected with vectors pcDNA3.1-S expressing the spike protein and pNL4-3.luc.RE bearing the luciferase reporter-expressing HIV-1 backbone. After 48 hours, the supernatants containing pseudotyped virus were harvested and filtered (0.22 μm pore size). Serial dilutions of antibodies P22A-1D1 and its three variants in DMEM supplemented with 10% fetal calf serum were incubated with pseudoviruses at 37°C for 1 h and then the mixtures were added to monolayer Huh-7 cells (10^4^ per well in 96-well plates). Twelve hours after infection, the culture medium was refreshed and then incubated for additional 48 hours. The luciferase activity was calculated for the detection of relative light units using the Bright-Glo™ Luciferase Assay System (Promega)

**Reference**

1. Ye, J., Ma, N., Madden, T.L. & Ostell, J.M. IgBLAST: an immunoglobulin variable domain sequence analysis tool. *Nucleic Acids Res* **41**, W34-40 (2013).

2. Gupta, N.T.*, et al.* Change-O: a toolkit for analyzing large-scale B cell immunoglobulin repertoire sequencing data. **31**, 3356-3358 (2015).

3. Schrodinger, LLC. The PyMOL Molecular Graphics System, Version 1.8. (2015).


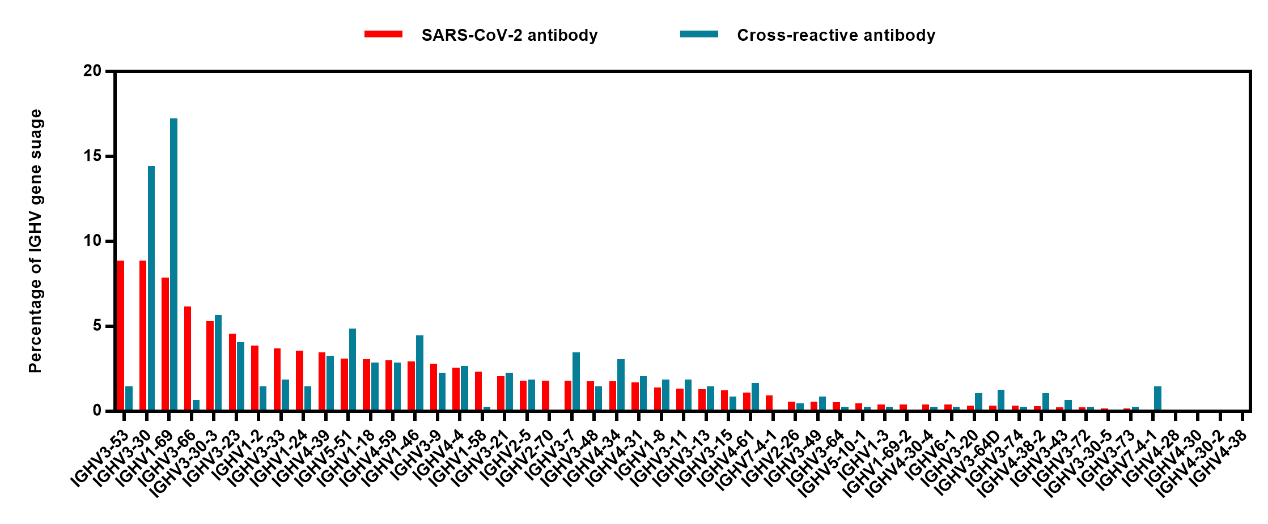


**Fig. S1. The IGHV gene usage distribution of SARS-CoV-2 binding antibodies**

**
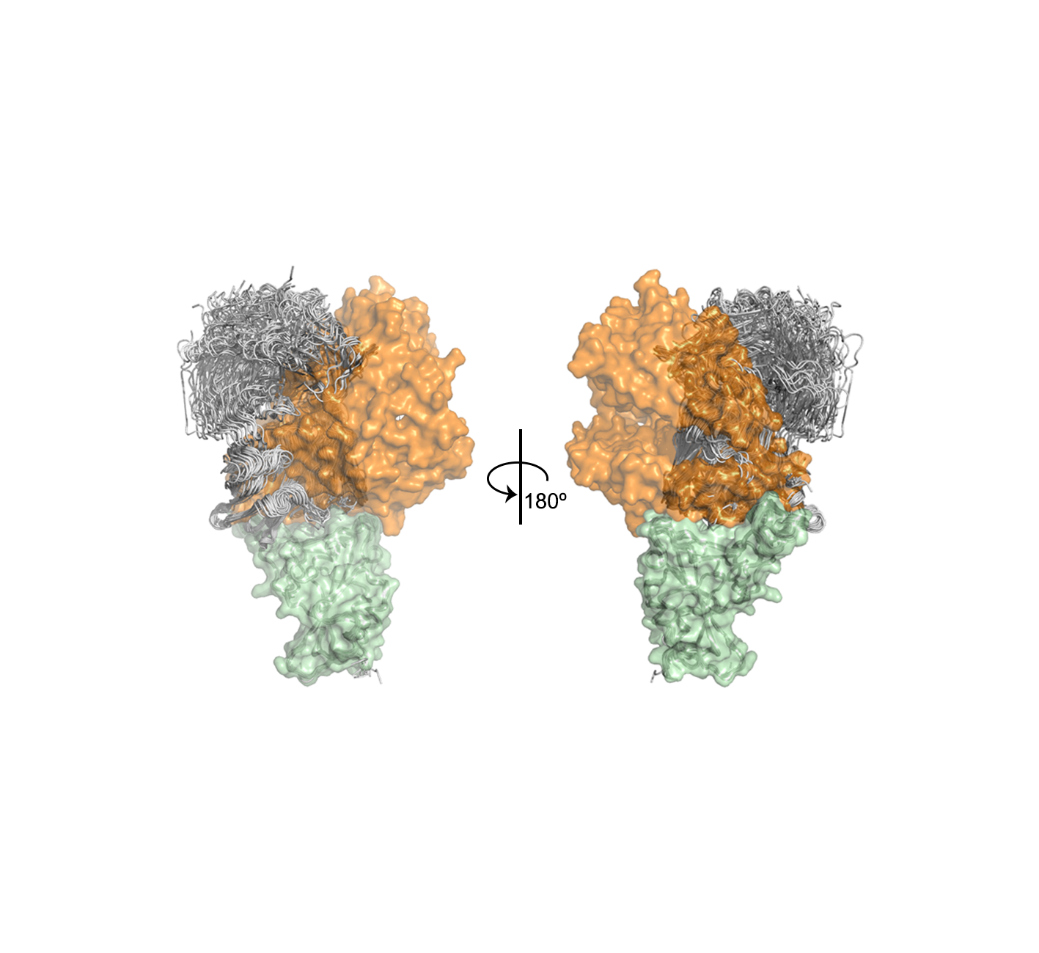
**

**Fig. S2. The binding mode of IGHV3-53/3-66 gene-encoded antibodies with RBD.**

Binding poses of IGHV3-53/3-66 antibodies (grey ribbon) and ACE2 (orange surface) on RBD (green surface and ribbon).


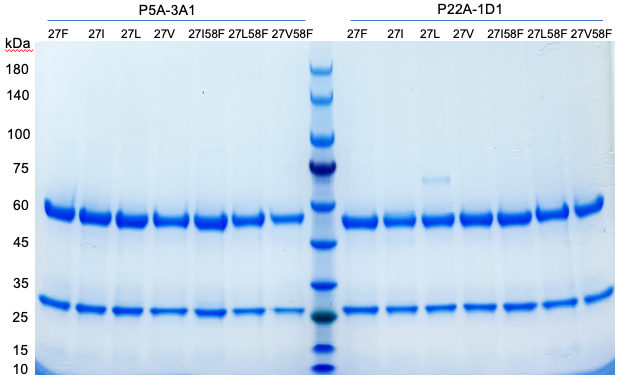


**
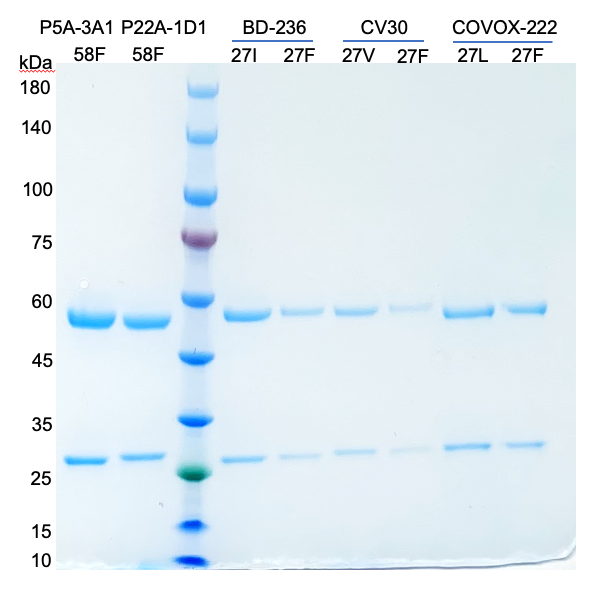
**

**Fig. S3. The purity and integrity of P5A-3A1, P22A-1D1, BD-236, CV30, COVOX-222 as well as their variants as determined by SDS-PAGE.**

**
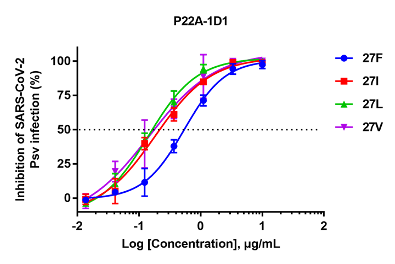
**

**
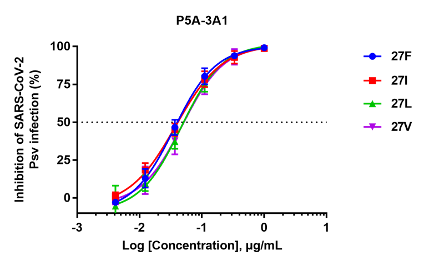
**

**Fig. S4 Neutralization of luciferase-encoding pseudotyped viruses with the spike protein of SARS-CoV-2 by mAbs of P22A-1D1, P5A-3A1 and their 27I, 27L and 27V variants.**

**Fig. S5. Binding kinetics of mAb BD-236, COVOX-222, CV30 and their reverse variants (mutate residue 27 to F) to SARS-CoV-2 RBD.**

**Fig. S6. Binding kinetics of mAb P5A-3A1, P22A-1D1 and their double mutations of Y58F and F27I/L/V to SARS-CoV-2 RBD.**

**Tab. S1 Lists of SARS-CoV-2 functionally germline antibodies.**

| **IGHV gene** | **Antibody** |
| --- | --- |
| IGHV3-53 | C099, P2B-1A10, Wang-C394, H712007+K711670, P5A-3A1, CC12.13, CC12.13, CC12.13, P22A-1D1, DH1205, CV38-183, COVA2-20 |
| IGHV3-30 | P17-A11, P5A-1B6, COVA2-34, CV2, CV7, CV41, CV43 |
| IGHV1-69 | C648, Wang-C431, Wang-C434, C548, Ehling_mab-82, C013 |
| IGHV3-66 | C617, COVA1-18, 2M-10B11 |
| IGHV3-30-3 | Wang-C474, H025794+K023710, H511600+K511320, H026009+K023830, H712154+K711779, DH1183, DH1162, DH1174, DH1175, COV2-2768, CV34 |
| IGHV3-23 | C207 |

**Tab. S2 Primers for antibodies’ variants construction.**

| **Primers** | **Sequences** |
| --- | --- |
| P22A-1D1-1F | attcggccacataggccgtct |
| P22A-1D1-H-I27-1R | tcatgtagttggaactcacggtaatgccacttgcggcacatgacagc |
| P22A-1D1-H-I27-2F | gctgtcatgtgccgcaagtggcattaccgtgagttccaactacatga |
| P22A-1D1-H-L27-1R | tcatgtagttggaactcacggttaagccacttgcggcacatgacagc |
| P22A-1D1-H-L27-2F | gctgtcatgtgccgcaagtggcttaaccgtgagttccaactacatga |
| P22A-1D1-H-V27-1R | tcatgtagttggaactcacggtaacgccacttgcggcacatgacagc |
| P22A-1D1-H-V27-2F | gctgtcatgtgccgcaagtggcgttaccgtgagttccaactacatga |
| P22A-1D1-2R | atgggcccttggtggaggctga |
| P22A-1D1-H-58F-1R | agaatcagcgtaaaaggtggatccgccaga |
| P22A-1D1-H-58F-2F | ctggcggatccaccttttacgctgattct |
| P5A-3A1-1F | attcggccacataggccgtct |
| P5A-3A1-H-I27-1R | tcatgtagtttgaggatacagtgattccgctggctgcgcatgagagt |
| P5A-3A1-H-I27-2F | actctcatgcgcagccagcggaatcactgtatcctcaaactacatga |
| P5A-3A1-H-L27-1R | tcatgtagtttgaggatacagtcaatccgctggctgcgcatgagagt |
| P5A-3A1-H-L27-2F | actctcatgcgcagccagcggattgactgtatcctcaaactacatga |
| P5A-3A1-H-V27-1R | tcatgtagtttgaggatacagtgactccgctggctgcgcatgagagt |
| P5A-3A1-H-V27-2F | actctcatgcgcagccagcggagtcactgtatcctcaaactacatga |
| P5A-3A1-H-58F-1R | ggaatcggcatagaaggtggaccctccgct |
| P5A-3A1-H-58F-2F | agcggagggtccaccttctatgccgattcc |
| P5A-3A1-2R | atgggcccttggtggaggctga |
| BD236-1F | attcggccacataggccgtct |
| BD236-1R | gtaattggagctcaccgtaaatccagaggcggcgcaggaaagcct |
| BD236-2F | aggctttcctgcgccgcctctggatttacggtgagctccaattac |
| BD236-2R | atgggcccttggtggaggctga |
| CV30-1F | attcggccacataggccgtct |
| CV30-1R | tgacatataattgctactcacaatgaatccggaagccgcacatga |
| CV30-2F | tcatgtgcggcttccggattcattgtgagtagcaattatatgtca |
| CV30-2R | atgggcccttggtggaggctga |
| COVOX-222-1F | attcggccacataggccgtct |
| COVOX-222-1R | tatagttgctgctgactgtaaagccagaagccgcaca |
| COVOX-222-2F | tgtgcggcttctggctttacagtcagcagcaactata |
| COVOX-222-2R | atgggcccttggtggaggctga |
